# Anaplastic transformation of papillary thyroid carcinoma in multiple lung metastases presenting with a malignant pleural effusion: a case report

**DOI:** 10.1186/1752-1947-8-460

**Published:** 2014-12-23

**Authors:** Tomoe Abe, Masaru Suzuki, Kaoruko Shimizu, Naofumi Shinagawa, Satoshi Oizumi, Yoshihiro Matsuno, Masaya Miyazaki, Mishie Tanino, Shinya Tanaka, Masaharu Nishimura

**Affiliations:** 18 First Department of Medicine, Hokkaido University School of Medicine, North 15 West 7, Kita-ku, Sapporo, 060-8638 Japan; 19 Department of Surgical Pathology, Hokkaido University Hospital, North 14 West 5, Kita-ku, Sapporo, 060-8648 Japan; 20 Department of Cancer Pathology, Hokkaido University Graduate School of Medicine, North 15 West 7, Kita-ku, Sapporo, 060-8638 Japan

**Keywords:** Anaplastic transformation, Lung metastasis, Malignant pleural effusion, Papillary thyroid carcinoma

## Abstract

**Introduction:**

Anaplastic transformation of well-differentiated papillary thyroid carcinoma at distant metastasis sites is rare. To the best of our knowledge, this is the first report of an autopsy case of anaplastic transformation of papillary thyroid carcinoma in multiple lung metastases presenting with a malignant pleural effusion.

**Case presentation:**

We report an autopsy case of a 61-year-old Japanese man with anaplastic transformation of papillary thyroid carcinoma with multiple lung metastases presenting with a malignant pleural effusion, which was difficult to diagnose by cytological examination before the autopsy. He presented with a 1-month history of progressive dyspnea, and examination of the left pleural effusion revealed a bloody exudate with an increase in thyroglobulin; however, malignant cells in the pleural fluid were negative for thyroglobulin.

**Conclusion:**

It is important to be aware that anaplastic transformation of differentiated thyroid carcinoma could develop in lung metastases and could be a cause of a malignant pleural effusion.

## Introduction

Papillary thyroid carcinoma is the most common malignant tumor of the thyroid and has a relatively favorable prognosis. However, anaplastic transformation of well-differentiated papillary thyroid carcinoma is considered rare and is associated with a very poor prognosis [1]. Anaplastic transformation of differentiated papillary thyroid carcinoma at distant metastasis sites is extremely rare. To date, to the best of our knowledge, only one case of anaplastic transformation of metastatic papillary thyroid carcinoma in the lung has been reported in the English-language literature, and that patient did not have a malignant pleural effusion [2]. Our present report is, to the best of our knowledge, the first description of an autopsy case of anaplastic transformation of papillary thyroid carcinoma in multiple lung metastases with a malignant pleural effusion, which was difficult to diagnose before the autopsy.

## Case presentation

**Figure 1:**
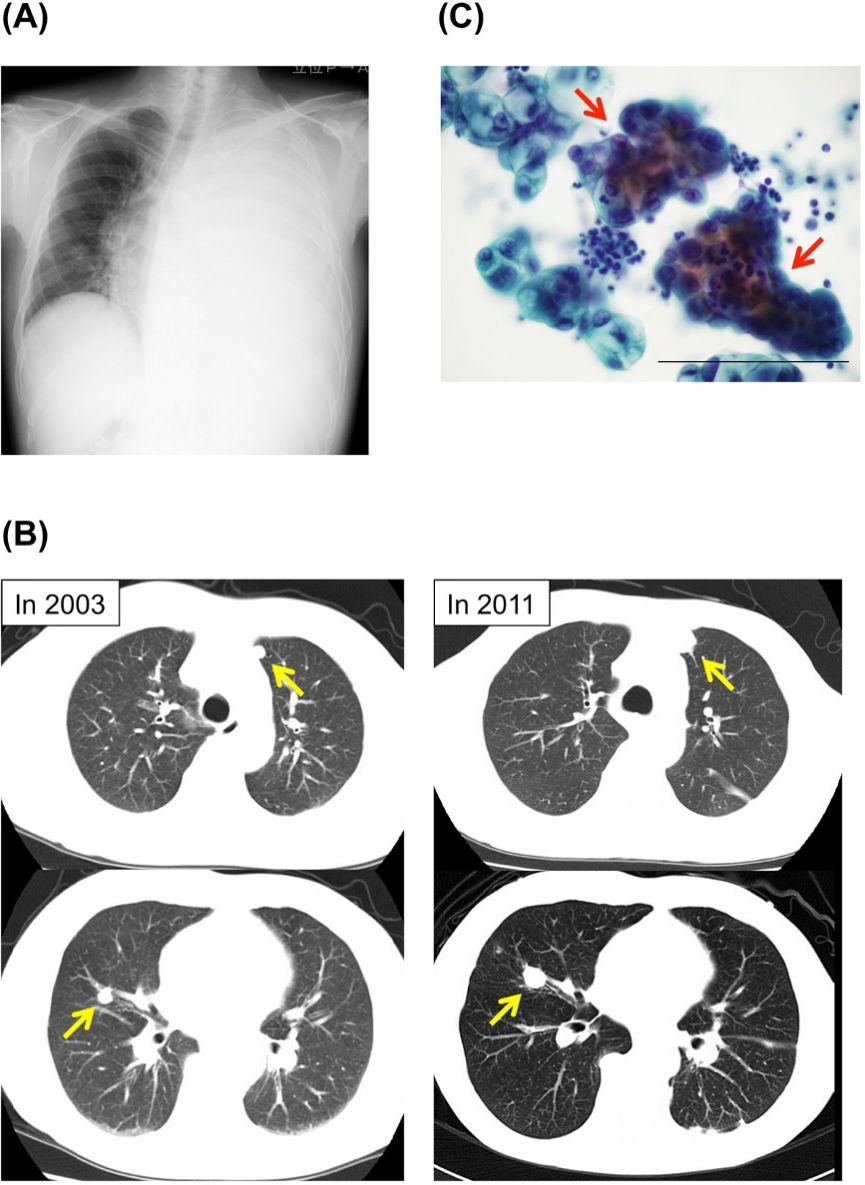
**Clinical findings of the case. (A)** Chest X-ray obtained on admission in 2011 shows a massive left-sided pleural effusion. **(B)** Multiple lung nodules (yellow arrows) are visible on computed tomographic scans obtained in 2003 (left images) and on admission in 2011 (right images, after drainage of pleural effusion). **(C)** Cytological findings of the pleural effusion. Cell clusters with peripherally located irregular nuclei (red arrows) are shown. Scale bar: 100μm.

A 61-year-old Japanese man who had a 1-month history of progressive dyspnea was referred to our hospital in April 2011. He had undergone a total thyroidectomy in 2001 for well-differentiated papillary thyroid carcinoma that showed no evidence of anaplastic transformation. He had then been treated with radioactive iodine-131 (^131^I) until 2003 for multiple lung nodules that were diagnosed as metastases of thyroid carcinoma. A chest X-ray obtained upon his admission to our hospital revealed a massive left-sided pleural effusion (Figure 1A). Multiple lung nodules had increased in size very slowly as visualized by computed tomography since 2003 (Figure 1B). Serum thyroglobulin and pro-gastrin-releasing peptide were elevated (93.2ng/mL and 75.2pg/mL, respectively), whereas other tumor markers, including carcinoembryonic antigen (CEA), carbohydrate antigen 19–9 (CA19-9), sialyl Lewis X and neuron-specific enolase, were all negative. A transbronchial biopsy of one of the lung nodules in the left upper lobe was attempted, but the biopsy instrument could not be properly inserted to the target lesion. Examination of the left pleural effusion by thoracentesis revealed bloody exudate (total protein 4.6g/dL, albumin 2.8g/dL, lactate dehydrogenase 191U/L) with an increase in thyroglobulin (198.9ng/mL). CEA, CA19-9 and adenosine deaminase were negative in the pleural fluid. The pleural fluid cytology was positive for adenocarcinoma (Figure 1C). A cell block made from the effusion also showed evidence of adenocarcinoma, which included papillary cell clusters with peripherally located irregular nuclei and cord-like structures, and the tumor cells were positive for thyroid transcription factor 1 (TTF-1) but negative for surfactant apoprotein A and thyroglobulin on immunohistochemistry. At that time, the origin of the malignant pleural effusion was considered not to be from papillary thyroid carcinoma but from primary lung adenocarcinoma, because of the pathological findings of the malignant pleural effusion and the appearance of a massive pleural effusion despite the very slow progressive nature of the lung nodules. The patient could not undergo any systemic chemotherapy, because his general condition worsened due to pneumonia and hemoptysis, and he died in October 2011.

**Figure 2:**
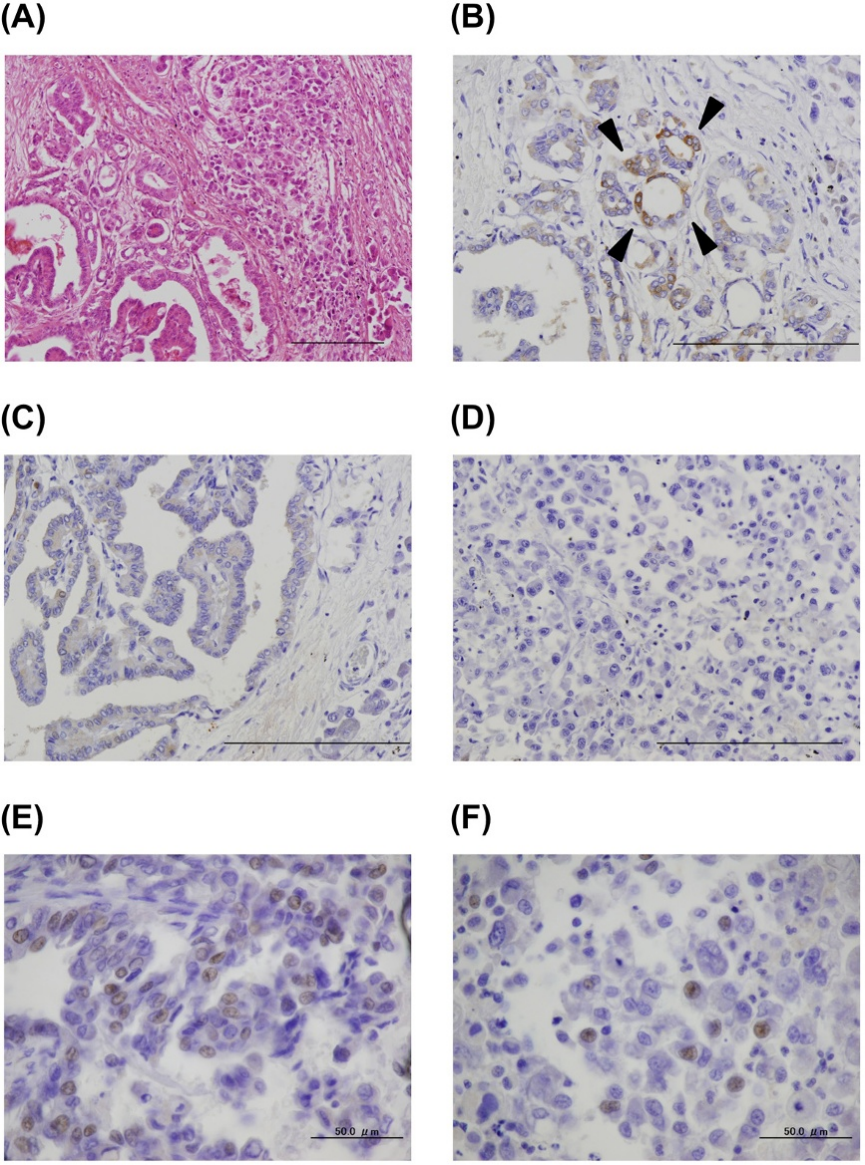
**Microscopic findings of the lung metastatic tumor at autopsy. (A)** Hematoxylin and eosin stain. A transitional zone between the differentiated papillary pattern (left side of the image) and the undifferentiated component (right side of the image) is shown. **(B, C, D)** Immunohistochemical staining for thyroglobulin. Differentiated papillary carcinoma positive (**B**, arrowheads) and negative **(C)** for thyroglobulin and an undifferentiated component negative for thyroglobulin **(D)** are shown. **(E, F)** Immunohistochemical staining for thyroid transcription factor 1. Differentiated papillary carcinoma positive for thyroid transcription factor 1 **(E)** and an undifferentiated component positive for thyroid transcription factor 1 **(F)** are shown. Scale bars: 200μm **(A-D)**, 50μm **(E, F)**.

During the autopsy, multiple nodules were found in both lungs, and some of the tumors in the left lung directly invaded the pericardium and the pleura. Furthermore, metastatic lesions were found in the peritoneum, liver, pancreas, spleen, both adrenal glands, para-aortic lymph nodes and bone marrow. The histologic examination revealed that the lung nodules in both lungs partly contained typical papillary thyroid carcinoma morphology with a papillary pattern, ground-glass nuclei and nuclear inclusions. However, the largest part of the tumors showed a pleomorphic and undifferentiated component, without follicular structures, that was negative for thyroglobulin. These two different histological areas showed transitional zones between them (Figure 2A). The differentiated tumor cells consisted of thyroglobulin-positive and thyroglobulin-negative components (Figures 2B and 2C), and the undifferentiated tumor cells were negative for thyroglobulin (Figure 2D). TTF-1 was partially positive in both differentiated and undifferentiated areas (Figures 2E and 2F), which was compatible with the pleural effusion specimens. Taken together, these findings suggested anaplastic transformation of papillary thyroid carcinoma in multiple lung metastases.

## Discussion

Anaplastic thyroid cancer is the most aggressive form of thyroid cancer and accounts for only 1% to 2% of all thyroid tumors [1]. The prognosis of anaplastic thyroid cancer is very poor, with a median survival of 4 to 12 months and a 5-year survival rate of 1.0% to 7.1% [3]. Although the pathogenesis of anaplastic thyroid cancer remains unclear, anaplastic transformation from pre-existing differentiated thyroid cancer has become a well-accepted process [3]. In fact, it has been reported that 23% to 90% of anaplastic thyroid cancer has an associated differentiated component [3]. Papillary thyroid carcinoma is the most common type of thyroid cancer associated with anaplastic transformation [1].

Anaplastic transformation most commonly occurs in the thyroid gland and regional lymph nodes, whereas anaplastic transformation of papillary thyroid carcinoma in several distant metastatic sites has been reported, including the submandibular space [4], retroperitoneum [5], liver [6], breast [7], shoulder [8] and lung [2]. In these cases and in the present case, it took 5 to 20 years for anaplastic transformation to develop after the primary diagnosis of papillary thyroid carcinoma. Therefore, it should be noted that anaplastic transformation can occur in metastatic sites even after long-term follow-up.

In our patient, it was difficult to diagnose anaplastic transformation of papillary thyroid carcinoma before the autopsy. In general, metastatic thyroid carcinoma is not considered in the differential diagnosis of malignant pleural effusion, because primary thyroid cancer is an extremely rare cause of pleural effusion. The prevalence of malignant pleural effusion in all thyroid cancers is reported to be 0.25% [9]. Moreover, in our patient, tumor cells in the pleural effusion were positive for TTF-1 but negative for thyroglobulin on immunohistochemistry, which made it even more difficult to identify the origin of the malignant pleural effusion as being the thyroid.

Cytomorphological studies have demonstrated that the tumor cells in the malignant pleural effusion with metastatic well-differentiated papillary thyroid carcinoma are immunoreactive for thyroglobulin, whereas anaplastic carcinoma is known for losing thyroglobulin expression [9]. In our patient, the tumor cells in the pleural effusion showed evidence of adenocarcinoma but were negative for thyroglobulin, suggesting that the cells were from thyroglobulin-immunonegative elements of papillary thyroid carcinoma. However, the pleural fluid in our patient contained high levels of thyroglobulin. Although the elevated level of thyroglobulin in the pleural fluid might just reflect high serum thyroglobulin levels, it has been reported that elevated pleural fluid thyroglobulin could be a potential biomarker for the diagnosis of metastatic thyroid cancer as a cause of pleural effusion [10].

The presence of transitional zones between the differentiated and undifferentiated components suggests the anaplastic transformation of pre-existing papillary thyroid carcinoma. Although the molecular pathogenesis of anaplastic transformation is not completely understood, some gene mutations, including the *BRAF*, *RAS*, *CTNNB1* (β-catenin), *TP53* and *PIK3CA*, has reportedly been associated with anaplastic carcinoma, and chromosomal abnormalities are also common [11]. In addition, ^131^I therapy has been considered to be associated with an increase in the probability of anaplastic transformation [6, 12, 13]. It has also been reported that insufficient accumulation of ^131^I in the metastatic sites was associated with development of anaplastic changes [14]. Post-operative ^131^I therapy was performed in five of seven cases of anaplastic transformation of papillary thyroid carcinoma in distant metastatic sites, including our patient [2, 5, 6, 8]. However, it remains unclear at this time whether ^131^I therapy is definitely associated with the pathogenesis of anaplastic transformation, so further clinical cases need to be examined.

## Conclusion

To the best of our knowledge, we report the first case of anaplastic transformation of papillary thyroid carcinoma in multiple lung metastases presenting with a malignant pleural effusion. It should be kept in mind that anaplastic transformation of differentiated thyroid carcinoma could develop in lung metastases and could also be a cause of malignant pleural effusion.

## Consent

Written informed consent was obtained from the patient’s next of kin for publication of this case report and any accompanying images. A copy of the written consent is available for review by the Editor-in-Chief of this journal.

## Abbreviations

*BRAF*: B-Raf proto-oncogene, serine/threonine kinase
CA19-9: Carbohydrate antigen 19–9
CEA: Carcinoembryonic antigen
*CTNNB1*: Catenin (cadherin-associated protein), β1, 88kDa
LDH: Lactate dehydrogenase
NSE: Neuron-specific enolase
*PIK3CA*: Phosphatidylinositol-4,5-bisphosphate 3-kinase, catalytic subunit α *RAS*, Rat sarcoma
*TP53*: Tumor protein p53
TTF-1: Thyroid transcription factor 1
